# Potential Use of Bisphosphonates in Invasive Extramammary Paget's Disease: An Immunohistochemical Investigation

**DOI:** 10.1155/2013/164982

**Published:** 2013-03-31

**Authors:** Taku Fujimura, Sadanori Furudate, Yumi Kambayashi, Setsuya Aiba

**Affiliations:** Department of Dermatology, Tohoku University Graduate School of Medicine, Sendai 980-8574, Japan

## Abstract

Invasive extramammary Paget's disease (EMPD) is relatively rare and is reported to be highly metastatic to lymph nodes or even other organs, including bone. Histologically, EMPD shows significant numbers of lymphocytes around the tumor mass, suggesting the possible development of novel immunomodulatory therapy for EMPD by targeting these infiltrating lymphocytes. Previously, bisphosphonates (BPs) were administered for the treatment of malignancy, especially osteolytic bone disease. Recent reports also suggested that BPs might have a direct antitumor effect through several pathways beyond their beneficial effect on bone metastasis. Among them, the abrogation of immunosuppressive cells, myeloid derived suppressor cells (MDSC), by BPs might be one of the optimal methods to induce an antitumor immune response both locally and at sites remote from the tumor. In this study, we employed immunohistochemical staining for immunosuppressive macrophages and cytotoxic T cells in the lesional skin of patients with noninvasive EMPD and those with invasive EMPD.

## 1. Introduction

Extramammary Paget's disease (EMPD) is a skin adenocarcinoma that generally occurs in the anogenital region [1]. It usually affects older patients, and the lesions commonly develop in the vulva, penis, scrotum, perineum, perianal area, umbilicus, and axilla [1]. Invasive EMPD, although relatively rare, is reported to be highly metastatic to lymph nodes (47.1%) or even other organs (17.6%), including bone (5.9%) [2]. Histologically, both noninvasive EMPD and invasive EMPD show significant numbers of lymphocytes around the tumor mass. 

The use of bisphosphonates (BPs) in malignancy, especially for osteolytic bone disease, has been increasing [3–5]. Recent reports suggested that BPs might have a direct antitumor effect beyond their beneficial effects on bone metastasis [3]. One of the possible explanations for the additional antitumor effects of BPs is that pharmacological inhibition of MMP9 by aminobisphosphonate decreases pro-MMP9 and VEGF in the serum and abrogates the suppressive function of immunosuppressive cells and induces the antitumor immune response both locally and at sites remote from the tumor [6]. In this study, we employed immunohistochemical staining for immunosuppressive macrophages and cytotoxic T cells in the lesional skin of patients with noninvasive EMPD and those with invasive EMPD.

## 2. Materials and Methods

### 2.1. Reagents

We used the following antibodies (Abs) for immunohistochemical staining: mouse monoclonal Abs for human CD8 (Dako A/S, Glostrup, Denmark), human granulysin (MBL LTD, Nagoya, Japan), anti-TIA1 Ab (Abcam, Cambridge, UK), antiperforin Ab (Kamiya Biomedical Company, Seattle, WA, USA), and human CD163 (Novocastra, UK), and rabbit polyclonal Abs for human MMP-9 (Abcam), human B7H1 (ProSci, Poway, CA, USA), and human arginase 1 (ARG1) (Life Span Bioscience, Seattle, WA). 

### 2.2. Tissue Samples and Immunohistochemical Staining

We collected archival formalin-fixed paraffin-embedded skin specimens from 5 patients with noninvasive EMPD and 5 patients with invasive EMPD treated at the Department of Dermatology at Tohoku University Graduate School of Medicine. We summarized these cases in Table 1. We defined EMPD by the typical clinical features and histological characteristics such as Paget's cells, defined as rounded cells that are devoid of intracellular bridges and have large nuclei and ample cytoplasm, seen in the epidermis. Invasive EMPD is histologically defined as Paget's cells infiltrating in the dermis. Immunohistochemical staining for both invasive and noninvasive EMPD is cytokeratin 7+, cytokeratin 20−, PAS+, and Alcian blue stain (AB)+ in all cases. The 5 noninvasive EMPD samples and 5 invasive EMPD samples were processed for single staining of CD8, granulysin, TIA1, perforin, CD163, MMP9, B7H1, or ARG1 as described previously [7–9].

### 2.3. Assessment of Immunohistochemical Staining

Staining of infiltrated lymphocytes was examined in more than 5 random, representative fields from each section. The number of immunoreactive cells was counted using an ocular grid of 1 cm^2^ at a magnification of 400x. Data are expressed as the mean ± standard deviation for Treg fractions in each skin disorder.

### 2.4. Statistical Analysis

For a single comparison of 2 groups, Student's *t*-test was used. The level of significance was set at *P* = 0.05.

## 3. Results

### 3.1. CD8, Granulysin, TIA-1, and Perforin in Invasive and NonInvasive EMPD

**Figure 1:**
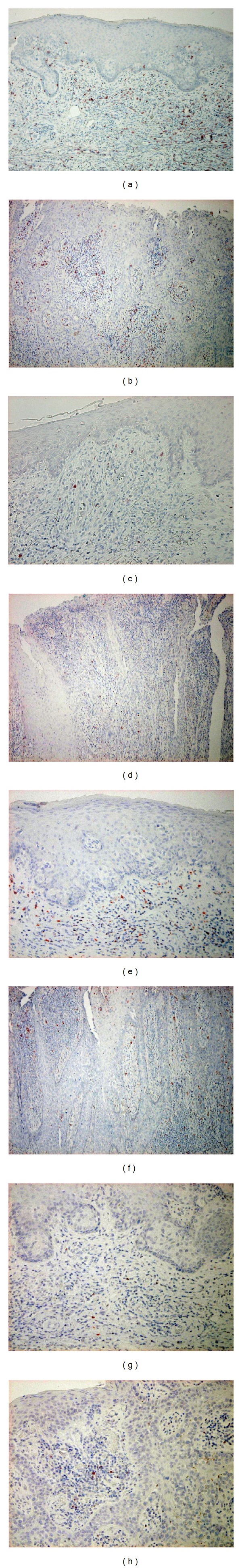
CD8, granulysin, TIA-1, and perforin in noninvasive and invasive EMPD. Paraffin-embedded tissue samples from patients with invasive and noninvasive EMPD were deparaffinized and stained using a combination of anti-CD8 Ab ((a) and (b)) and anti-granulysin Ab ((c) and (d)), anti-TIA-1 Ab ((e) and (f)) or antiperforin Ab ((g) and (h)). Noninvasive EMPD: (a), (c), (e), and (g); invasive EMPD: (b), (d), (f), and (h). Original manifestation: ×200. Sections were developed with liquid permanent red.

**Figure 2:**
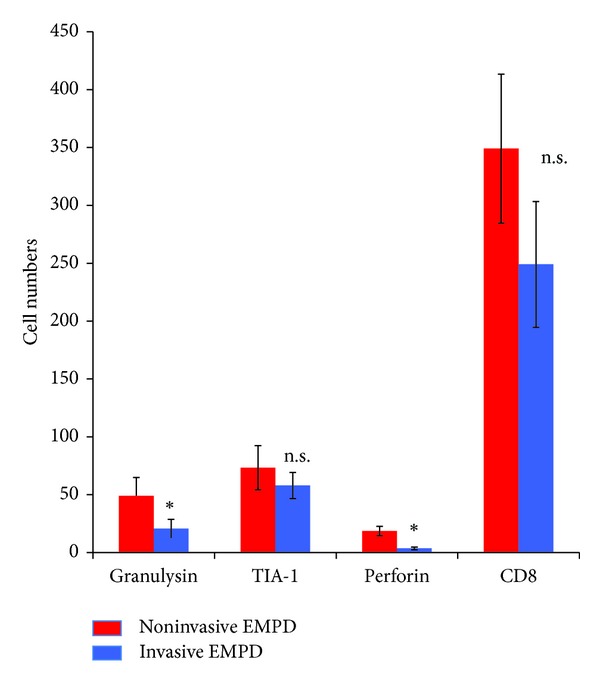
Summary of the numbers of CD8+ cells, granulysin bearing cells, TIA-1+ cells, and perforin+ cells in noninvasive and invasive EMPD. Five representative fields of each section were selected from each group of EMPD. The number of immunoreactive cells was counted using an ocular grid of 1 cm^2^ at a magnification of 400x. The data are expressed as the means SD of the numbers in each area. Stars: *P* < 0.05.

First, to compare the profiles of tumor-infiltrating cytotoxic T lymphocytes between invasive and noninvasive EMPD, we employed immunohistochemical staining for CD8 (Figures 1(a) and 1(b)), granulysin (Figures 1(c) and 1(d)), TIA-1 (Figures 1(e) and 1(f)), and perforin (Figures 1(g) and 1(h)). The numbers of granulysin^+^ cells and perforin^+^ cells were significantly lower in invasive EMPD than in noninvasive EMPD (granulysin: invasive EMPD versus noninvasive EMPD; 20.7 ± 8.1 versus 49.0 ± 15.9) (perforin: invasive EMPD versus noninvasive EMPD; 3.7 ± 1.2 versus 18.7 ± 4.0) (*P* < 0.05). In contrast, there was no significant difference in the numbers of CD8^+^ and TIA-1^+^ cells in the peritumoral areas of invasive and noninvasive EMPD (CD8: invasive EMPD versus noninvasive EMPD; 249 ± 54.4 versus 349 ± 64.3) (TIA-1: invasive EMPD versus noninvasive EMPD; 58.0 ± 11.4 versus 73.3 ± 19.1). We summarize the numbers of cytotoxic cells in Figure 2. As we previously described, the ratio of Foxp3^+^ cells to CD3, CD4 and CD25 positive cells was significantly lower in invasive EMPD [7].

### 3.2. CD163, B7H1, MMP-9, and ARG1 in Invasive EMPD

**Figure 3:**
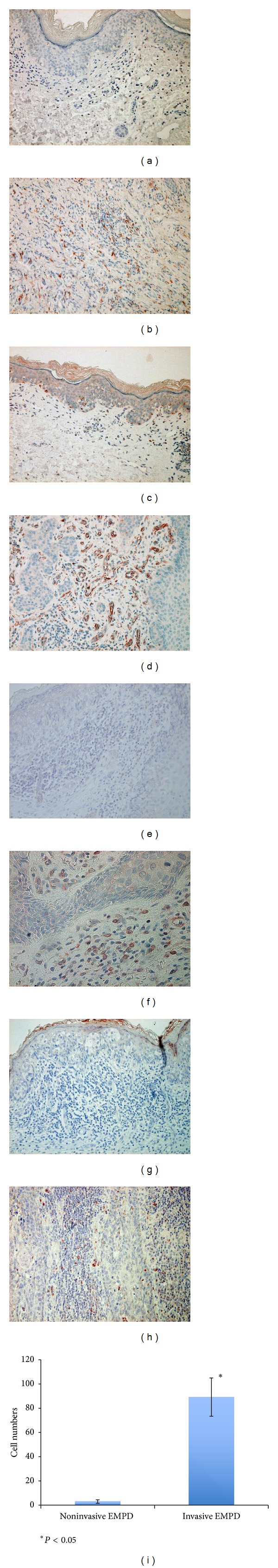
Anti-CD163, anti-MMP-9, anti-B7H1, and anti-ARG1 antibody staining of noninvasive and invasive EMPD. Paraffin-embedded tissue samples from patients with non-invasive EMPD ((a), (c), (e), and (g)) and invasive EMPD ((b), (d), (f), and (h)) were deparaffinized and stained with the anti-CD163 Ab ((a) and (b)), anti-MMP-9Ab ((c) and (d)), anti-B7H1-Ab ((e) and (f)), or anti-ARG1 Ab ((g) and (h)). Sections were developed with liquid permanent red. (CD163: staining for macrophages, MMP-9 staining, and B7H1 for macrophages and endothelial cells) ((a)–(e), (g) (h): ×200, (f): ×400). Five representative fields of each section were selected from each group of EMPD. The number of CD163^+^ cells in invasive and noninvasive EMPD was counted using an ocular grid of 1 cm^2^ at a magnification of 400x and summarized. The data are expressed as the means SD of the numbers in each area. Stars: *P* < 0.05.

To further investigate the profiles of immunosuppressive cells around the tumors in invasive and noninvasive EMPD, we performed immunohistochemical staining of CD163 (Figures 3(a) and 3(b)) as well as the functional markers for M2 macrophages, MMP-9 (Figures 3(c) and 3(d)), B7H1 (Figures 3(e) and 3(f)), and ARG1 (Figures 3(g) and 3(h)). Only in invasive EMPD were dense CD163^+^ macrophages detected throughout the dermis. Interestingly, the expression of MMP-9, B7H1, and ARG1 was observed at the same areas as the CD163^+^ macrophage-infiltrating areas of invasive EMPD (Figures 3(d), 3(f), and 3(h)), whereas few MMP-9, B7H1, and ARG1 expressing cells were detected in noninvasive EMPD (Figures 3(c), 3(e), and 3(g)). We summarized the number of CD163^+^ cells in Figure 3(i). The numbers of CD163^+^ cells were significantly higher in invasive EMPD than in noninvasive EMPD (Figure 3(i)) (CD163: invasive EMPD versus noninvasive EMPD; 3.0 ± 1.4 versus 89.2 ± 15.8) (*P* < 0.05).

## 4. Discussion

Immunosuppressive macrophages, M2 macrophages, and myeloid derived suppressor cells (MDSC), together with Tregs, were reported to promote an immunosuppressive environment in the tumor-bearing host [10–12]. Alternatively activated macrophages, M2 macrophages, have an important role in the response to parasite infection, tissue remodeling, angiogenesis, and tumor progression [13]. MDSCs are a heterogeneous population of cells that promote an immunosuppressive environment in tumor-bearing hosts [10]. In human, MDSCs are a less defined and phenotypically heterogeneous group of cells that have only immunosuppressive activities in common. Among them, arginase 1 (ARG1) is reported as a marker for polymorphonuclear MDSCs [10]. In this aspect, MDSCs in human are translated CD163^+^, ARG1^+^, and alternatively activated, tumor-associated macrophages (TAM) [11].

MMP-9 is a stromal factor that regulates the mobilization of hematopoietic stem cells from the bone marrow niche by solubilizing the membrane-bound form of c-KitL [14]. Because it remodels the extracellular matrix and promotes the sprouting and growth of new blood vessels by making VEGF available to the VEGFR-2/flk receptor on endothelial cells, MMP-9 is a linchpin in tumor progression [14]. Actually, several reports revealed that the expression of MMP-9 on tumors was correlated with the progression or prognosis of several skin tumors such as malignant melanoma, squamous cell carcinoma, basal cell carcinoma, mycosis fungoides, extramammary Paget's disease, and angiosarcoma [7, 9, 14–19]. In addition, other reports described that the expression of MMP-9 on immunosuppressive macrophages in the tumor microenvironment contributed to tumor invasion and metastasis [6, 7, 9, 19, 20]. In aggregate, these reports suggest that increased numbers of MMP-9^+^ cells around the tumor might be connected with CD163^+^ M2 macrophages and contribute to the poor prognosis of the tumor-bearing host.

**Figure 4:**
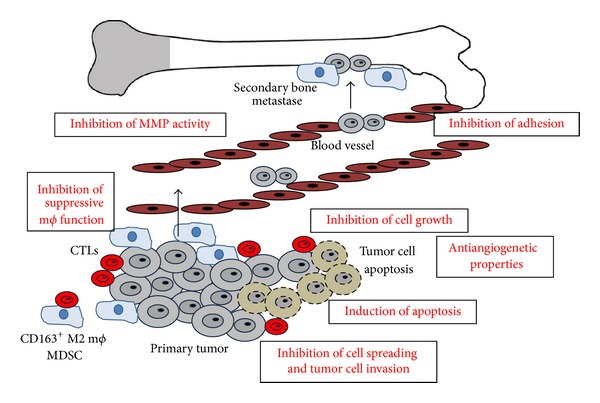
Schematic representation of antitumor effect of BPs. BPs might suppress the progression of EMPD as follows: induction of tumor apoptosis, inhibition of the function of immunosuppressive macrophages, inhibition of tumor adhesion, inhibition of tumor invasion, and inhibition of MMP activity.

The use of bisphosphonates (BPs) in malignancy, especially osteolytic bone disease, has been increasing [3–5]. Recent reports suggested that BPs might have a direct antitumoral effect beyond their beneficial effect on bone metastasis [3]. Various investigations have demonstrated the synergistic, antiproliferation effect of BPs with conventional chemotherapeutic drugs in vitro (Figure 4) [4, 5]. Indeed, Fehm et al. reported that the antitumor effect of BPs for breast cancer cells in vitro is equal or even superior to those of chemotherapeutic drugs, such as DTX [5]. In addition, from the immunological point of view, it was reported that pharmacological inhibition of MMP9 by aminobisphosphonate decreased pro-MMP9 and VEGF in the serum and abrogated the induction of MDSC in the tumor microenvironment [6]. In aggregate, the administration of BPs in tumor-bearing hosts might abrogate the suppressive function of immunosuppressive cells, such as MDSC and M2 macrophages, and induce the antitumor immune response at the local site of the tumor. Indeed, in this report, we employed immunohistochemical staining for invasive and noninvasive EMPD and revealed that both invasive and noninvasive EMPD contains substantial numbers of cytotoxic T cells (CD8, granulysin, TIA1, and perforin). Interestingly, only invasive EMPD possessed substantial numbers of CD163^+^ M2 macrophages and MMP-9^+^ cells, B7H1^+^ cells, and ARG1^+^ cells around the tumor, whereas few CD163^+^ M2 macrophages, MMP-9^+^ cells, B7H1^+^ cells, and ARG1^+^ cells were observed in noninvasive EMPD.

## 5. Conclusion

Our data suggest that the administration of BPs for patients with invasive EMPD by targeting the immunosuppressive macrophages might be effective not only for the prevention of bone metastasis, but also for the prevention of the progression of the disease both locally and at sites remote from the tumor. Since we did not directly assess the suppressive function of these infiltrating M2 macrophages or cytotoxic T cells, further analysis of the mechanisms underlying this phenomenon will be necessarily to confirm our limited observation.

## Figures and Tables

**Table 1:** Summary for 10 cases of invasive or noninvasive EMPD. We summarized the treatment, clinical stage, and prognosis of invasive or noninvasive EMPD.

	Ages/sex	Radical therapy	Stage	Prognosis
Noninvasive EMPD				
Case 1	82/M	Surgical resection	Stage I	Complete remission
Case 2	70/M	Surgical resection	Stage I	Complete remission
Case 3	92/M	Surgical resection/radiation (60G)	Stage I	Complete remission
Case 4	81/M	Surgical resection	Stage I	Complete remission
Case 5	70/M	Surgical resection	Stage I	Complete remission
Invasive EMPD				
Case 6	69/M	Surgical resection/radiation (60G)	Stage IV	Dead by multiple metastasis
Case 7	78/M	Surgical resection/lymphadenectomy	Stage III	Complete remission
Case 8	78/F	Surgical resection	Stage II	Complete remission
Case 9	80/M	Surgical resection	Stage II	Complete remission
Case 10	82/M	Surgical resection/lymphadenectomy	Stage III	Complete remission
